# From LAL-D to MASLD: Insights into the role of LAL and Kupffer cells in liver inflammation and lipid metabolism

**DOI:** 10.1016/j.bbalip.2024.159575

**Published:** 2024-10-31

**Authors:** Ivan Bradić, Katharina B. Kuentzel, Anita Pirchheim, Silvia Rainer, Birgit Schwarz, Michael Trauner, Martin R. Larsen, Nemanja Vujić, Dagmar Kratky

**Affiliations:** a Gottfried Schatz Research Center, Molecular Biology and Biochemistry, Medical University of Graz, Graz, Austria; b Department of Biochemistry and Molecular Biology, University of Southern Denmark, Odense, Denmark; c Hans Popper Laboratory of Molecular Hepatology, Division of Gastroenterology and Hepatology, Department of Internal Medicine III, Medical University of Vienna, Vienna, Austria; d BioTechMed-Graz, Graz, Austria

**Keywords:** Lysosomal acid lipase deficiency, Kupffer cell depletion, Clodronate, Metabolic dysfunction-associated steatotic liver, disease, Liver fibrosis

## Abstract

Metabolic dysfunction-associated steatotic liver disease (MASLD) is a prevalent liver pathology worldwide, closely associated with obesity and metabolic disorders. Increasing evidence suggests that macrophages play a crucial role in the development of MASLD. Several human studies have shown an inverse correlation between circulating lysosomal acid lipase (LAL) activity and MASLD. LAL is the sole enzyme known to degrade cholesteryl esters (CE) and triacylglycerols in lysosomes. Consequently, these substrates accumulate when their enzymatic degradation is impaired due to LAL deficiency (LAL–D). This study aimed to investigate the role of hepatic LAL activity and liver-resident macrophages (i.e., Kupffer cells (KC)) in MASLD. To this end, we analyzed lipid metabolism in hepatocyte-specific (hep)Lal−/− mice and depleted KC with clodronate treatment. When fed a high-fat/high-cholesterol diet (HF/HCD), hepLal −/− mice exhibited CE accumulation and an increased number of macrophages in the liver and significant hepatic inflammation. KC were depleted upon clodronate administration, whereas infiltrating/proliferating CD68-expressing macrophages were less affected. This led to exacerbated hepatic CE accumulation and dyslipidemia, as evidenced by increased LDL-cholesterol concentrations. Along with proteomic analysis of liver tissue, these findings indicate that hepatic LAL-D in HF/HCD-fed mice leads to macrophage infiltration into the liver and that KC depletion further exacerbates hepatic CE concentrations and dyslipidemia.

## 1 Introduction

Metabolic dysfunction-associated steatotic liver disease (MASLD), previously known as non-alcoholic fatty liver disease, encompasses a broad spectrum of disorders characterized by hepatic lipid accumulation in the absence of alcohol abuse or infectious etiology [1]. The primary risk factors for the development of MASLD include obesity, insulin resistance, and type 2 diabetes mellitus [1]. From 1991 to 2019, the global prevalence of MASLD exhibited a gradual increase, rising from 21.9 % to 37.3 % [2]. Hepatic steatosis is a less severe form of the disease, characterized by lipid deposition in the liver without hepatocellular damage. In contrast, metabolic dysfunction-associated steatohepatitis (MASH) is associated with hepatic steatosis, liver inflammation, and hepatocyte ballooning without or with fibrosis [1,3]. Lipidomic analyses revealed an accumulation of triacylglycerol (TG) and free cholesterol (FC) without a similar increment in cholesteryl esters (CE) in both steatosis and MASH patients [4]. These pathological conditions may eventually lead to cirrhosis and ultimately to the development of hepatocellular carcinoma [3,5]. Up to 30 % of MASLD patients develop MASH [5]. All histological stages of MASLD are linked to a marked increase in overall mortality, which is directly proportional to the severity of MASH and the stage of fibrosis [6].

Numerous studies have demonstrated a correlation between decreased lysosomal acid lipase (LAL) activity in the blood [7–14] or liver [13] and MASLD. Reduced circulating LAL activity was found to be independently associated with non-cirrhotic MASLD (patients with steatosis or MASH without fibrosis), alcoholic cirrhosis, and MASH-related cirrhosis, but not with non-cirrhotic alcoholic liver disease. In contrast, patients with metabolic syndrome but normal blood LAL activity were less prone to develop MASLD [14]. LAL is the only known enzyme responsible for the hydrolysis of CE [15] and TG in lysosomes at acidic pH [16]. Various mutations of the LAL-encoding *LIPA* gene have been associated with the loss of LAL enzymatic activity and the development of LAL deficiency (LAL–D) [17]. Low to no residual LAL activity is linked to hepatosplenomegaly, liver steatosis and fibrosis, adrenal calcification, and malabsorption, which ultimately leads to death in infancy [18–20]. Patients with up to 10 % residual LAL activity develop late-onset LAL–D. Although the International LAL-D Registry (NCT01633489), which enrolled 228 patients, did not explicitly mention changes in life expectancy [21], another study reported that >50 % of these patients died of liver failure by the age of 21 years due to hepatic steatosis, cirrhosis, and dyslipidemia [22]. Given the unspecific clinical manifestations of liver disease presented by patients with LAL–D, there is a risk of being misdiagnosed as MASLD [22].

The progressive pathogenesis of LAL-D has been extensively studied in LAL-deficient (Lal−/−) mice, which develop hepatosplenomegaly, liver steatosis, and dyslipidemia [23–25], similar to the symptoms observed in LAL-D patients. To study the role of LAL in liver pathology, hepatocyte-specific Lal−/− (hepLal−/−) mice may be more suitable since the severe phenotype observed in Lal−/− mice affects numerous organs [26]. In contrast to the mild phenotype of hepLal−/− mice fed a chow diet, feeding a high-fat/high-cholesterol diet (HF/HCD) results in increased liver weight, liver fibrosis, and infiltration of liver macrophages [26]. This histopathology is comparable to that of MASH mouse models [27–30]. In addition, hepLal−/− mice develop liver injury as early as 10 weeks of HF/HCD, whereas C57BL/6 J mice develop profound hepatic steatosis, inflammation, fibrosis, and other features that resemble human MASH only after 30 weeks of HF/HCD feeding [28]. One proposed mechanism for the development of MASH is the accumulation of cholesterol/CE crystals in hepatocytes, which activates Kupffer cells (KC), leading to immune cell infiltration and liver inflammation [27]. KC have been shown to affect the progression of MASLD [31–33], but their role in the context of decreased hepatic LAL activity in MASLD remains elusive.

The present study aimed to investigate the impact of decreased hepatic LAL activity on liver pathology under HF/HCD feeding and to assess the contribution of KC to the development of a MASH-like phenotype by KC depletion in the presence and absence of hepatic LAL. Our findings demonstrate that decreased hepatocyte LAL activity promotes liver inflammation and fibrosis, whereas KC depletion alters lipid metabolism in hepLal−/− livers without further aggravating liver injury.

## 2 Material and methods

### 2.1 Animals

The experiments were performed with age-matched female hepLal−/− mice (C57BL/6 J background) and their corresponding Lipa^flox/flox^ (WT) littermates [26]. The mice were housed in a clean and temperature-controlled environment (22 °C ± 1 °C) with unlimited access to chow diet (Altromin 1324, Lage, Germany) and water, following a regular 12-h light/12-h dark cycle. Female hepLal−/− and control mice used for the KC depletion study received HF/HCD (60 % caloric intake from fat plus 1 % cholesterol; D12492 (II) mod. plus 1 % Cholesterol; Ssniff, Soest, Germany) for 15 weeks, starting at 7 to 13 weeks of age. Male and female hepLal−/− and control mice used for hepatocyte isolation were fed either chow or HF/HCD for 20 weeks. The animal experiments were conducted in accordance with the European Directive 2010/63/EU, complied with national laws, and were approved by the Austrian Federal Ministry of Education, Science and Research, Vienna, Austria (BMBWF-66.010/0106-V/3b/2019, BMWFW-66.010/0109-WF/V/3b/2015).

### 2.2 Depletion of KC by clodronate treatment

After 14 weeks of HF/HCD feeding, hepLal−/− and control mice received intraperitoneal (i.p.) injections of three doses of clodronate liposomes (10 μL/g body weight, clodronate concentration ~ 5 mg/mL) or control (PBS-containing) liposomes (Liposoma BV, Amsterdam, Netherlands) 48 h apart. Two days after the third dose, mice were fasted for 4 h before blood sampling, sacrifice, and organ collection.

### 2.3 Hepatocyte and non-parenchymal cells isolation

Hepatocytes were isolated following a previously described protocol with minor modifications [34]. In brief, mice were anesthetized with a terminal anesthetic mixture containing ketamine and xylazine diluted in PBS. All buffers used were kept at approximately 36 °C to ensure proper digestion and cell viability. The livers were perfused with a perfusion buffer (0.5 mM EDTA and 25 mM HEPES in Hanks’ Balanced Salt Solution (HBSS) without Ca^2+^ and Mg^2+^) via the inferior vena cava, and the liquid was drained by cutting the portal vein. The livers were digested by perfusion for 10 min with digestion buffer (1 mg/mL Liberase TM Research Grade (Sigma-Aldrich, St. Louis, MO) and 25 mM HEPES in HBSS with Ca^2+^, Mg^2+^, and phenol red). The digested livers were carefully dissected and transferred into Dulbecco’s Modified Eagle Medium (DMEM; 1 g/L glucose and sodium pyruvate; Thermo Fisher Scientific, Waltham, MA). Cells were squeezed from the digested liver into DMEM, and the suspension was filtered through a 70-μm cell strainer. The plate was rinsed once with 10 mL DMEM, and the suspension was again filtered through a 70-μm cell strainer. The suspension was centrifuged at 50 x*g* for 3 min, and the supernatant was transferred to another tube, while the hepatocyte pellet was resuspended in 10 mL DMEM, centrifuged at 20 x*g* for 3 min, and the pellet was stored at −80 °C as hepatocyte-enriched fraction. The supernatant was centrifuged again at 50 x*g* for 3 min, the supernatant was collected and centrifuged at 650 x*g* for 5 min. The pellet was washed with 10 mL DMEM, centrifuged at 650 x*g* for 5 min, and stored at −80 °C as the nonparenchymal cells-enriched (NPC) fraction.

### 2.4 RNA isolation and quantitative real-time PCR (qRT-PCR)

Total RNA was isolated from the liver and perigonadal white adipose tissue (pWAT) using TriFast (Peqlab, Erlangen, Germany) following the manufacturer’s protocol. Isolated RNA (1 μg) was then reverse transcribed using the High Capacity cDNA Reverse Transcription Kit (Applied Biosystems, Carlsbad, CA). qRT-PCR analyses were performed using GoTaq® qPCR Mastermix (Promega, Fitchburg, WI) and the Bio-Rad CF X96 real-time PCR system (Bio-Rad Laboratories, Hercules, CA). mRNA expression was analyzed in duplicate, normalized to the expression of *cyclophilin A*, and calculated using the 2^−ΔΔCT^ method. The primer sequences are listed in Supplementary Table S1.

### 2.5 Quantification of protein concentration and Western blotting experiments

The liver tissue from the larger lobe was sonicated in lysis buffer (250 mM sucrose, 100 mM potassium phosphate, 1 mM EDTA, pH 7) supplemented with 1 mM dithiothreitol (DTT) and protease inhibitor cocktail (P8340, 1:1000; Sigma–Aldrich, St. Louis, MO) for 4 ×10 s each on ice. The lysates were then centrifuged at 20,000 x*g* and 4 °C for 10 min, and the protein concentration in the supernatant was determined using the DC™ Protein Assay Kit (Bio-Rad Laboratories, Hercules, CA). Fifty micrograms of protein were separated by SDS-PAGE and transferred to a PVDF membrane, which was then incubated overnight at 4 °C with the following primary antibodies: CD68 (SM1550P, 1:1000; Ori-Gene Technologies, Rockville, MD), CLEC4F (PA5–47396, 1:1000; Thermo Fisher Scientific, Waltham, MA), LAL (PA5–27346, 1:1000; Thermo Fisher Scientific), β-actin (sc-47,778, 1:10,000; Santa Cruz, Heidelberg, Germany), and α-tubulin (NB100–690; 1:5000, Novus Biologicals LLC, Centennial, CO). Secondary anti-mouse (P0260; 1:500; Dako, Glostrup, Denmark), anti-rat (31,470; 1:2000; Thermo Fisher Scientific), and anti-goat (sc-2922; 1: 5000; Santa Cruz, Heidelberg, Germany) antibodies were incubated with the membrane for 2 h at room temperature (RT). Proteins were visualized using ECL (Bio-Rad Laboratories) and the ChemiDoc™ Imaging System (Bio-Rad Laboratories).

### 2.6 H&E and Sirius red staining

Fresh liver tissue was fixed in 4 % PBS-buffered paraformaldehyde for 24 h, embedded in paraffin, and sectioned (5 μm). The tissue sections were deparaffinized and then incubated with hematoxylin (Sigma-Aldrich, St. Louis, MO) for 10 min, followed by eosin for 1 min. Picro-Sirius red staining (ScyTek Laboratories, Inc., Logan, UT) was performed for 1 h at RT on sections that had been incubated with hematoxylin for 8 min and washed for 2 min in warm tap water. The sections were dehydrated (2× dH_2_O, 75 % EtOH, 98 % EtOH, xylene/EtOH, xylene), and mounted in a resinous medium.

### 2.7 Immunofluorescence

Paraffin-embedded liver tissue was deparaffinized, washed with 0.1 % Tween-20 in PBS (PBS-T), and sectioned (5 μm). The sections were incubated in PBS-T/10 % goat serum and anti-mouse CD16/CD32 monoclonal antibody (1:1000; 14–0161-85; Thermo Fisher Scientific, Waltham, MA) for 30 min. Primary rabbit anti-LAL antibody (1:200; PA5–27346, Thermo Fisher Scientific) and rat anti-CD68 (1:250; SM1550PS; OriGene Technologies, Rockville, MD) were diluted in PBS-T containing 10 % goat serum and incubated with the liver sections overnight at 4 °C. The samples were washed with PBS-T prior to incubation with the secondary antibodies (1:500; goat anti-rabbit IgG (H + L) cross-adsorbed secondary antibody, Alexa Fluor™ 594 (red) and goat anti-rat IgG (H + L) cross-adsorbed secondary antibody, Alexa Fluor™ 488 (green)) in PBS-T containing 10 % goat serum for 90 min at RT. The samples were washed with PBS-T, stained with DAPI for 5 min, and mounted with Vectashield Antifade Mounting Medium (Vector Laboratories, Newark, CA). The liver sections were imaged with an Olympus BX63 microscope.

### 2.8 Hepatic lipid extraction and quantification

The liver tissue from the larger lobe was processed as described in Section 2.5, and lipids from the lysates containing 1 mg of protein were extracted using the Folch method. TG, total cholesterol (TC), and FC concentrations were measured using enzymatic kits (DiaSys, Holzheim, Germany) according to the manufacturer’s guidelines. CE concentrations were calculated by subtracting FC from TC.

### 2.9 Plasma parameters

Plasma was isolated by centrifugation of freshly drawn EDTA blood for 7 min at 5200 x *g* and 4 °C. TG, TC, FC, and CE concentrations were determined as described in Section 2.8. Lipid distribution in lipoprotein particles was determined by measuring TG and TC concentrations in lipoprotein fractions after separation of 200 μL pooled plasma by fast protein liquid chromatography (FPLC; Pharmacia P-500, Uppsala, Sweden) equipped with a Superose 6 column. Plasma from lithiumheparinized blood was isolated by centrifugation at 5200 x*g* and 4 °C for 7 min and used for the measurement of alanine aminotransferase (ALT) and aspartate aminotransferase (AST) on Fuji Dri-Chem NX500 (FUJIFILM Holdings Corporation, Tokyo, Japan) with F1512 and F1511 slides for ALT and AST, respectively.

### 2.10 White blood cell counts

White blood cell counts from freshly drawn blood in EDTA-coated tubes were determined using the V-Sight Vet Hematology Analyzer (A. Menarini Diagnostics, Florence, Italy) according to the manufacturer’s protocol.

### 2.11 TG and CE hydrolase activity assays

TG hydrolase (TGH) and CE hydrolase (CEH) activities were measured at neutral (pH 7) and acidic (pH 4.5) conditions as described previously [35] with minor modifications. Briefly, proteins were extracted from a portion of pWAT and liver tissue using lysis buffer supplemented with 1 mM DTT and protease inhibitor cocktail, as described in Section 2.5. CEH activities at pH 7 and pH 4.5 were determined using a substrate containing 200 μM cholesteryl oleate and 0.04 μCi cholesteryl [1-^14^C]-oleate (Amersham Biosciences, Piscataway, NJ) per sample solubilized in 455 μM mixed micelles of phosphatidyl-choline and phosphatidylinositol (3:1). TGH activities at pH 4.5 were determined using 300 μM triolein and 0.5 μCi [9,10-^3^H(N)]-triolein (Perkin Elmer, Waltham, MA) per sample solubilized in 455 μM of the above-described mixed micelles. The substrate for TGH pH 7 activity was solubilized in 45 μM of the micelles. Fifty micrograms of protein were mixed with the appropriate substrate and diluted in either 100 mM citrate buffer (pH 4.2) or in 100 mM potassium phosphate buffer (pH 7). Assays were performed by incubating the samples at 37 °C for 1 h before stopping the reaction and measuring and calculating the activity, as recently described [25].

### 2.12 Total collagen assay

The total collagen assay kit based on the alkaline hydrolysis to yield free hydroxyproline (#ab222942; Abcam, Cambridge, UK) was utilized to determine collagen levels in the livers by following the manufacturer’s protocol with minor modifications. Briefly, the liver tissue was homogenized and sonicated in 100 μL dH_2_O per 10 mg of tissue, treated with 100 μL of 10 N NaOH, and boiled at 120 °C for 1 h. The boiled lysates were neutralized with 100 μL of 10 N HCL, vortexed, and centrifuged at 10,000 x*g* for 5 min. An aliquot of 10 μL was evaporated and redissolved in 100 μL of an oxidation mixture for 20 min. Subsequently, 50 μL of a developer solution was added and incubated at 37 °C for 5 min. Before sealing the plate and incubating the mixture at 65 °C for 45 min, the DMAB concentrate solution was added. The collagen levels were determined by measuring the absorbance at 562 nm and are represented in arbitrary units (AU).

### 2.13 Sample preparation for mass spectrometry (MS)-based proteomic and measurement

Thirty milligrams of pulverized and homogenized liver tissue were lysed with 1.4-mm ceramic beads (OMNI International, Inc., Kennesaw, GA) in 50 mM HEPES/5 % sodium deoxycholate (SDC) (pH 8.5), supplemented with 1× cOmplete™, EDTA-free Protease Inhibitor Cocktail and PhosSTOP phosphatase inhibitors (Sigma–Aldrich, St. Louis, MO). Afterwards, the samples were diluted to a final concentration of 2.5 % SDC in 50 mM HEPES (pH 8.5) and lysed by sonication for 4× 30 s at 60% amplitude. The samples were denatured by incubation at 99 °C and 700 rpm for 5 min. After centrifugation at 20,000 x*g* for 20 min, the infranatant was transferred into a new low-binding tube, and the protein concentration was determined using the Pierce™ BCA Protein Assay Kit (Thermo Fisher Scientific, Waltham, MA). Per sample, 20 μg of protein were reduced and alkylated with 10 mM DTT and 20 mM iodoacetamide, respectively. The proteins were digested overnight at 37 °C with LysC (0.04 activity units per mg of protein) and trypsin (5 %). The following day, an additional 1 % trypsin was added, and the samples were incubated for 2 h at 37 °C. Half of the digested proteins were transferred to another tube, and SDC was precipitated with 2 % trifluoroacetic acid (TFA). After centrifugation at 20,000 x*g* for 5 min, the supernatant was purified using C18 stage tips (Thermo Fisher Scientific). Peptides were eluted from the C18 stage tips with 65 % acetonitrile in 0.1 % TFA, vacuum-dried, resuspended in 50 μL of 0.1 % formic acid, and shaken at 2500 rpm for 15 min. Peptide concentrations were measured using the NanoPhotometer N60 (IMPLEN, Munich, Germany), and 200 ng of peptides were loaded onto EvoTips (Evosep, Odense, Denmark) following the manufacturer’s protocol. The peptides were then separated using the “30 samples per day” method on a 15-cm EV1106 Endurance Column packed with 150 μM C18 beads (1.9 μm) (Evosep) using an Evosep ONE HPLC connected to a timsTOF Pro 2 mass spectrometer (MS) (Bruker Daltonics, Bremen, Germany). The MS was operated in the dia-PASEF mode with a CaptiveSpray ionization source. MS data were collected between 100 and 1700 *m*/*z*. TIMS settings were set as: 1/K0 start 0.6 Vs/cm^2^; 1/K0 end 1.6 Vs/cm^2^; Ramp time 100 ms: Accumulation time 100 ms. Ion mobility was calibrated using 622.0290, 922.0098, and 1221.9906 Tuning Mix ES-TOF. Dia-PASEF settings included: dia-PASEF cycles of 1.7 s; 1/K0 from 0.66 to 1.4 Vs/cm^2^; 26 mass steps per cycle.

### 2.14 Proteomic data quantification and bioinformatics

Protein quantification was performed using DIA-NN (1.9) [36,37] by searching the raw data against the Mouse Uniprot reviewed FASTA file downloaded on July 01, 2024. The following parameters were used for precursor ion generation: FASTA digest for library-free search/library generation; deep learning-based spectra, retention time, and ion mobility spectra prediction; trypsin digestion with 2 missed cleavages; N-terminal methionine excision and carbamidomethylation on cysteine were set as fixed modifications; a maximum of 2 variable modifications were allowed, including oxidation on methionine and acetylation on the N-terminus; peptide length range was set to 7–30 amino acids; precursor charge range was set to 1–4; precursor *m*/*z* range was 300–1800; fragment ion m/z range was 200–1800. The precursor false discovery rate (FDR) was set to 1 %. Mass accuracy and MS1 accuracy were set to 15 ppm and 20 ppm, respectively. Peptidoforms, match between runs, heuristic protein inference, and exclusion of shared spectra were enabled. Protein inference was performed based on genes. The rest of the parameters were kept at default settings.

Data were analyzed with Perseus (2.0.10.0) [34] and Jupyter Notebook with Python 3.11.5. One of 5 WT+ Clod and 2 of 7 hepLal−/− + Clod samples measured were excluded due to poor data quality or sample preparation-related issues. The data were filtered for at least 70 % valid values in the entire dataset. Protein intensities were log_2_ transformed and missing values were imputed using MissForest algorithm in Jupyter Notebook utilizing the packages Pandas, Numpy, StandardScaler (sklearn.preprocessing), and MissForest. The data were median-normalized and the statistical significance was determined using 2-way ANOVA in Perseus. FDR correction was calculated in Jupyter Notebook utilizing Pandas and Numpy packages with a Benjamini-Hochberg test, and the significance threshold was set <5 %. Z-scored data were used for principal component analysis in Perseus. The heatmap with significantly changed proteins between WT and hepLal−/− samples was generated with Euclidean clustering in Perseus. A KEGG PATHWAY analysis was performed with the obtained clusters utilizing the Fisher exact test with a Benjamini-Hochberg FDR correction of 2 %. The top 20 enriched pathways in hepLal−/− compared to WT samples and vice versa were visualized in Jupyter Notebook using the packages Pandas, Numpy, Seaborn, Matplotlib.pyplot, Re, and Two-SlopeNorm from matplotlib.colors. Other heatmaps for significantly changed proteins were generated in Jupyter Notebook using Seaborn, Matplotlib.pyplot, Pandas, Numpy, Linkage, and Dendrogram from scipy.cluster.hierarchy.

The mass spectrometry proteomic data have been deposited to the ProteomeXchange Consortium via the PRIDE [38] partner repository with the dataset identifier “PXD056343”.

### 2.15 Statistical analysis

The data are presented as mean ± SD. Statistically significant differences were calculated using GraphPad Prism 10.3.1 software, with comparisons made using 2-way ANOVA followed by Tukey post-hoc test. *p < 0.05, **p ≤ 0.01, ***p ≤ 0.001 indicate statistical significance for comparisons between different genotypes within the same treatment group (WT vs hepLal−/− (PBS) or WT vs hepLal−/− (clodronate)); ^#^p < 0.05, ^##^p ≤ 0.01, ^###^p ≤ 0.001 indicate statistical significance for comparisons between different treatment groups within the same genotypes (PBS vs clodronate (WT) or PBS vs clodronate (hepLal−/−)). For the comparison between only WT PBS vs hepLal−/− PBS and hepLal−/− PBS vs hepLal−/− Clod, 1-way ANOVA followed by Bonferroni post-hoc test was used. A 3-way ANOVA was used to compare the effects of cell type (NPCs and hepatocytes), diet (chow and HF/HCD), and genotype (WT and hepLal−/−).

## 3 Results

### 3.1 Increased liver and spleen weights and macrophage infiltration in hepLal−/− mice after HF/HCD feeding

**Fig. 1:**
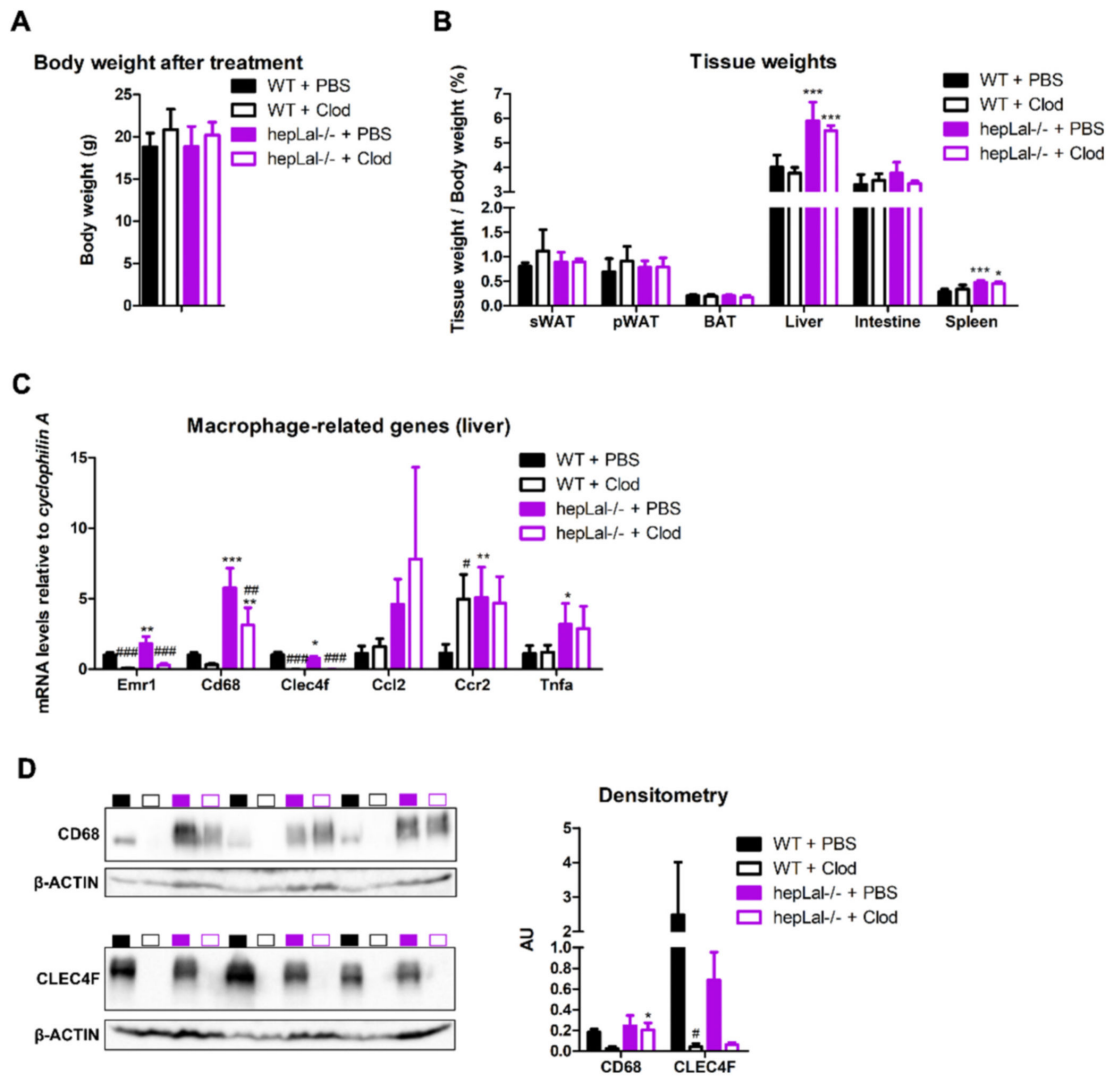
Hepatosplenomegaly and an increased number of macrophages in hepLal−/− mice fed a HF/HCD. (A) Body weight (n = 5–6) and (B) weight of subcutaneous white adipose tissue (sWAT), perigonadal WAT (pWAT), brown adipose tissue (BAT), liver, intestine, and spleen of 4-h fasted WT and hepLal−/− mice injected i.p. with PBS- or clodronate (Clod)-liposomes (n = 4–6). (C) mRNA expression of macrophage-related genes in the liver of WT and hepLal−/− mice (n = 3–7). (D) Western blots of CD68 and CLEC4F protein expression and their densitometric analyses in livers of WT + PBS (black box), WT + Clod (black border), hepLal−/− + PBS (magenta box), and hepLal−/− + Clod (magenta border) normalized to B-ACTIN and represented as arbitrary units (AU) (n = 3). Statistically significant differences were calculated by 2-way ANOVA followed by Tukey post-hoc test. *p < 0.05, **p ≤ 0.01, ***p ≤ 0.001 for comparisons between different genotypes with the same treatment (WT + PBS vs hepLal−/− + PBS and WT + Clod vs hepLal−/− + Clod); ^#^p < 0.05, ^##^p ≤ 0.01, ^###^p ≤ 0.001 for comparisons between different treatments of the same genotypes (WT + PBS vs WT + Clod and hepLal−/− + PBS vs hepLal−/− + Clod). Data represent mean values + SD. (For interpretation of the references to color in this figure legend, the reader is referred to the web version of this article.)

To induce a MASLD-like phenotype, we challenged female hepLal−/− and control mice with HF/HCD, which resulted in a comparable increase in body weight in both genotypes (Fig. S1A). After 14 weeks of HF/HCD feeding, the animals received three doses of clodronate or PBS-containing liposomes via i.p. injection and were euthanized 48 h after the third dose to investigate the consequences of KC depletion. Clodronate treatment had no effect on body weight (Fig. 1A) or on the weights of subcutaneous white adipose tissue (sWAT), perigonadal WAT (pWAT), brown adipose tissue (BAT), liver, small intestine, and spleen (Fig. 1B) in either genotype. Liver and spleen weights, however, were increased in hepLal−/− mice regardless of clodronate treatment (Fig. 1B). Increased mRNA expression of the macrophage markers *Emr1* and *Cd68* indicated a greater macrophage infiltration/proliferation in the livers of PBS liposome-injected hepLal / compared with WT mice (Fig. 1C). The expression of the monocyte chemoattractant *Ccl2* tended to be elevated and the expression of its receptor (*Ccr2*) and the proinflammatory cytokine *Tnfa* was significantly increased in hepLal−/− livers. The efficacy of the clodronate treatment was confirmed by the reduced expression of the KC marker *Clec4f* and of *Emr1* in both WT and hepLal−/− livers. *Cd68* mRNA expression was also strongly decreased in the livers of both genotypes after clodronate treatment, although it remained higher in hepLal−/− compared to WT mice (Fig. 1C), suggesting that the circulating monocytes may have had sufficient time to repopulate due to their relatively short lifespan in the blood, ranging from 20 h to 2 days [39]. The protein expression of CD68 and CLEC4F corresponded to their mRNA expression (Fig. 1D). These results indicated that liver inflammation and macrophage infiltration were increased in HF/HCD-fed hepLal−/− mice, and that clodronate was effective in reducing liver macrophages in both WT and hepLal−/− mice.

### 3.2 Clodronate treatment reduces mRNA expression of fibrotic genes in HF/HCD-fed hepLal−/− mice

**Fig. 2:**
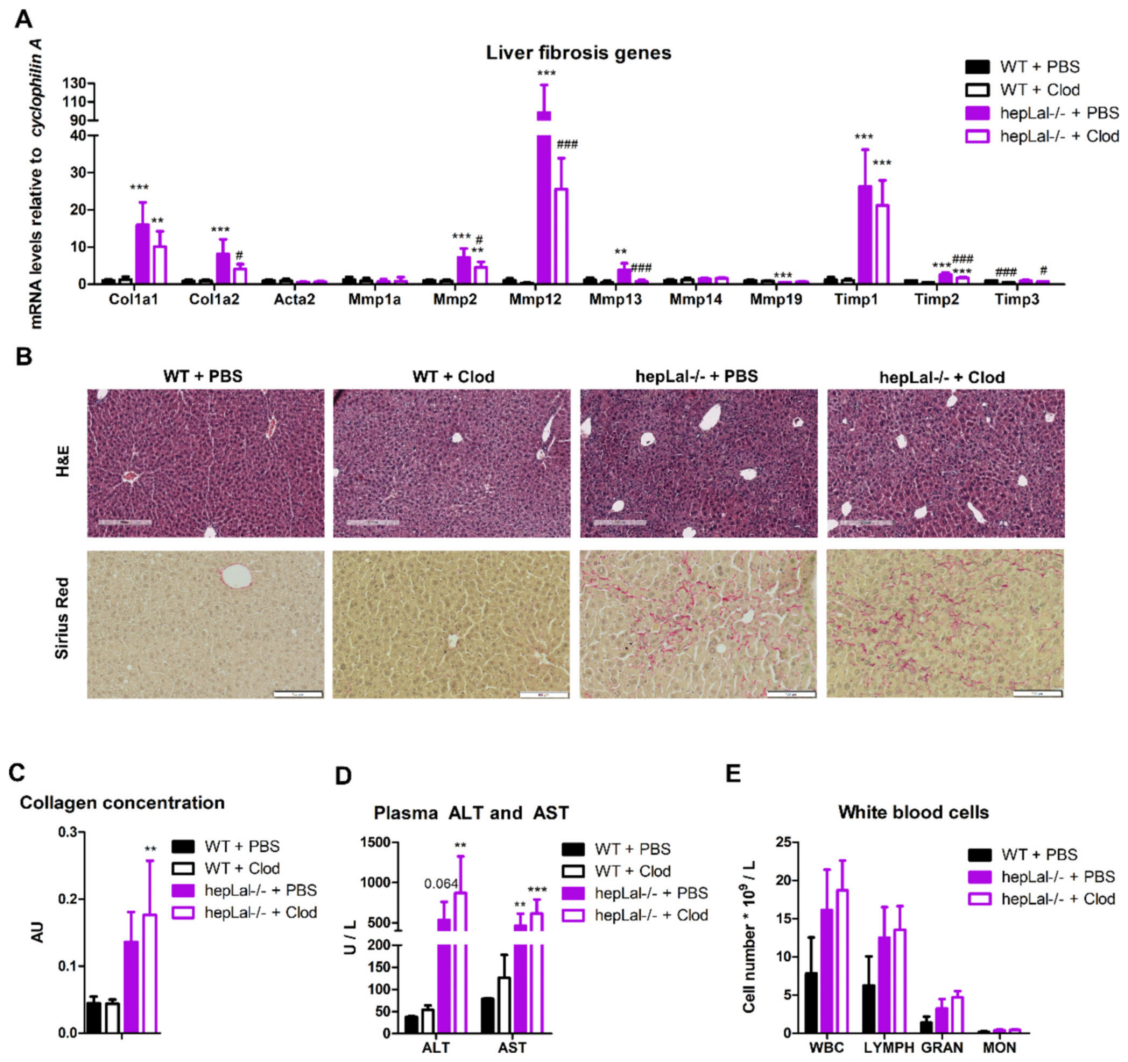
HF/HCD-fed hepLal−/− mice are more prone to liver injury than WT mice independent of KC depletion. (A) mRNA expression of fibrosis-related genes in livers of 4-h fasted WT and hepLal−/− mice injected i.p. with PBS- or clodronate (Clod)-liposomes (n = 4–7). (B) H&E (scale bar, 200 μm) and Sirius red staining (scale bar, 100 μm) of liver sections. (C) Hepatic collagen concentrations (n = 3–5). (D) Plasma alanine aminotransferase (ALT) and aspartate aminotransferase (AST) concentrations (n = 4–6). (E) White blood cell (WBC), lymphocyte (LYMPH), granulocyte (GRAN), and monocyte (MON) counts (n = 3–4). (A, C, D) Statistically significant differences were calculated by 2-way ANOVA followed by Tukey post-hoc test. (E) Statistically significant differences were calculated by 1-way ANOVA followed by Bonferroni post-hoc test. **p ≤ 0.01 and ***p ≤ 0.001 for comparisons between different genotypes with the same treatment (WT + PBS vs hepLal−/− + PBS and WT + Clod vs hepLal−/− + Clod); ^#^p < 0.05 and ^###^p ≤ 0.001 for comparisons between different treatments of the same genotypes (WT + PBS vs WT + Clod and hepLal−/− + PBS vs hepLal−/− + Clod). (For interpretation of the references to color in this figure legend, the reader is referred to the web version of this article.)

Given the pivotal role of KC and monocyte-derived macrophages in MASLD progression [40], we assessed the impact of hepatic macrophage depletion on the mRNA expression of markers of fibrosis. We observed a significant upregulation of the liver fibrosis markers *Col1a1* and *Col1a2* in hepLal−/− compared with WT livers (Fig. 2A), with clodronate treatment significantly reducing *Col1a2* mRNA expression in hepLal−/− livers. The mRNA expression of the majority of genes involved in fibrogenesis and fibrolysis, including metalloproteinase (*Mmp*)*2, Mmp12, Mmp13*, metallopeptidase inhibitor (*Timp*)*1*, and *Timp2*, was elevated in hepLal−/− compared with WT livers, and clodronate treatment decreased their expression in the livers of hepLal−/− mice (Fig. 2A). *Mmp19* expression was reduced in the livers of PBS-treated hepLal−/− compared to WT mice, whereas smooth muscle actin α2 (*Acta2*), *Mmp1a*, and *Mmp14* expression remained unaltered. *Timp3* was the sole MMP inhibitor whose expression was decreased by clodronate treatment in the livers of WT and hepLal−/− mice (Fig. 2A). The presence of liver fibrosis in hepLal−/− mice was confirmed by H&E, Sirius red staining, and collagen quantification, regardless of KC depletion, whereas livers of WT mice did not exhibit any liver fibrosis (Fig. 2B and C). Plasma AST levels were markedly increased in hepLal−/− mice regardless of treatment, whereas ALT levels tended to be elevated in hepLal−/− mice, but only reached significance in clodronate-treated animals (Fig. 2D). Clodronate did not alter circulating ALT and AST levels, indicating that KC or their depletion in hepatocyte-specific LAL-D have a minor effect on liver injury. Loss of LAL in hepatocytes also tended to increase the number of circulating white blood cells (WBC), lymphocytes, granulocytes, and monocytes. However, these cell counts remained unaffected by clodronate treatment (Fig. 2E). Collectively, these data suggest that loss of LAL in hepatocytes may promote liver injury and increase systemic inflammation.

### 3.3 KC contribute to CE degradation in livers of hepLal−/− mice

**Fig. 3:**
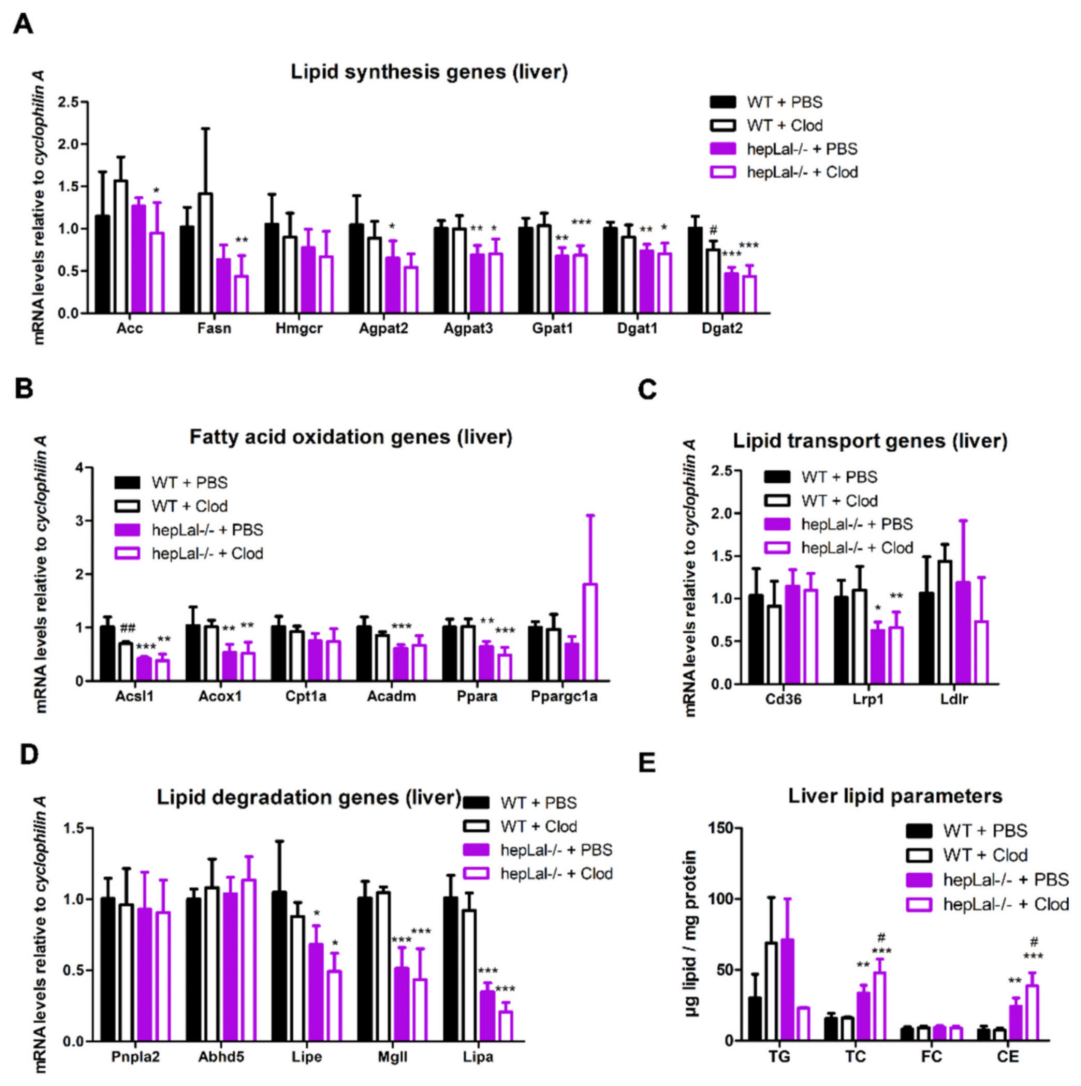
Altered liver lipid metabolism in HF/HCD-fed hepLal−/− mice. Livers were isolated from 4-h fasted WT and hepLal−/− mice injected i.p. with PBS- or clodronate (Clod)-liposomes. mRNA expression of genes involved in (A) lipid synthesis (n = 5–7), (B) fatty acid oxidation (n = 4–7), (C) lipid transport (n = 4–7), and (D) lipid degradation (n = 4–7). (E) Quantification of triacylglycerol (TG), total cholesterol (TC), free cholesterol (FC), and cholesteryl ester (CE) concentrations (n = 3–4). Statistically significant differences were calculated by 2-way ANOVA followed by Tukey post-hoc test. *p < 0.05, **p ≤ 0.01, ***p ≤ 0.001 for comparisons between different genotypes with the same treatment (WT + PBS vs hepLal−/− + PBS and WT + Clod vs hepLal−/− + Clod); ^#^p < 0.05 and ^##^p ≤ 0.01 for comparisons between different treatments of the same genotypes (WT + PBS vs WT + Clod and hepLal−/− + PBS vs hepLal−/− + Clod).

To examine the consequences of LAL-D and KC depletion on hepatic lipid homeostasis, we analyzed the mRNA expression of multiple genes related to lipid metabolism. The expression of genes involved in TG synthesis (*Agpat2, Agpat3, Gpat1, Dgat1, Dgat2*) was reduced in the livers of untreated hepLal−/− mice, and clodronate treatment reduced the expression of *Acc, Fasn, Agpat3, Gpat1, Dgat1*, and *Dgat2* compared with WT controls (Fig. 3A). *Hmgcr* mRNA expression was unaffected by genotype and treatment. The mRNA expression of several genes involved in fatty acid oxidation (*Acsl1, Acox1, Ppara*) was downregulated in hepLal−/− livers irrespective of treatment (Fig. 3B). The administration of clodronate reduced *Acsl1* mRNA expression in WT mice, whereas *Cpt1a* and *Ppargc1a* were unchanged in both genotypes (Fig. 3B).

Unaltered *Cd36* and *Ldlr* but decreased *Lrp1* expression indicated reduced uptake of chylomicron remnants and VLDL in the livers of hepLal−/− compared with WT mice, regardless of treatment (Fig. 3C). The expression levels of the cytosolic lipases *Lipe* (encoding hormonesensitive lipase) and *Mgll* (encoding monoglyceride lipase) were lower in PBS- and clodronate-injected hepLal−/− mice, whereas the expression of *Pnpla2* (encoding adipose triglyceride lipase) and its co-activator *Abhd5* were unchanged (Fig. 3D). As expected, *Lipa* mRNA expression was markedly decreased in the livers of hepLal−/− mice and tended to be further reduced by clodronate treatment (Fig. 3D), most likely due to the diminished number of LAL-expressing KC. The elevated TC and CE concentrations in hepLal−/− livers were further increased by clodronate treatment (Fig. 3E). Since clodronate treatment did not affect TC and CE levels in WT livers (Fig. 3E), this result indicates that CD68-positive macrophages are essential for CE degradation in livers that lack LAL specifically in hepatocytes.

### 3.4 HF/HCD restored LAL activity in hepLal−/− livers

**Fig. 4:**
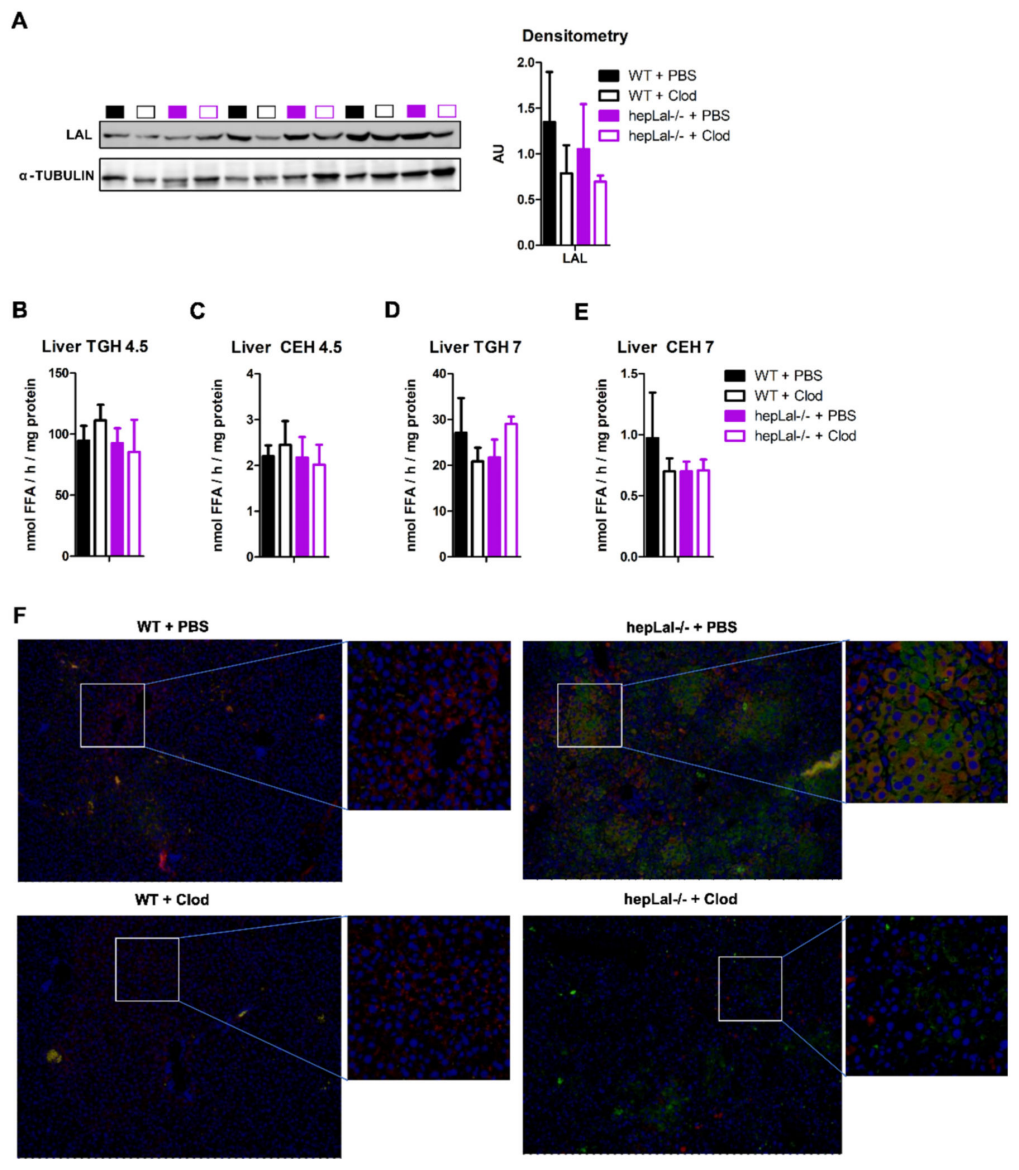
Unchanged LAL protein expression and activity in HF/HCD-fed hepLal−/− mice. Livers were isolated from 4-h fasted WT and hepLal−/− mice injected i.p. with 3 doses of PBS- or clodronate (Clod)-liposomes. (A) Western blot of LAL and densitometric analyses in livers of WT + PBS (black box), WT + Clod (black border), hepLal−/− + PBS (magenta box), and hepLal−/− + Clod (magenta border) normalized to α-tubulin and represented as arbitrary units (AU) (n = 3). (B, D) Hepatic TG hydrolase (TGH) and (C, E) CE hydrolase (CEH) activities at (B, C) pH 4.5 (n = 4–6) and (D, E) pH 7 (n = 4–6). (F) Immunofluorescence staining for LAL (red) and CD68 (green), nuclei are stained for DAPI (blue); magnification, 10×. (For interpretation of the references to color in this figure legend, the reader is referred to the web version of this article.)

Although *Lipa* mRNA expression was decreased in the livers of hepLal−/− mice (Fig. 3D), LAL protein expression (Fig. 4A) as well as TG and CE hydrolase activities at pH 4.5 were unchanged (Fig. 4B, C). As expected, TG and CE hydrolase activities at pH 7 remained comparable in the livers of both genotypes (Fig. 4D, E). These results suggest that LAL released from apoptotic or newly infiltrating macrophages contributes to LAL expression and activity in the livers of hepLal−/− mice. It is worth noting that acid TG and CE hydrolase activities were significantly decreased in the livers of chow diet-fed hepLal−/− compared with WT mice [41], demonstrating the lack of LAL activity in the livers of hepLal−/− mice. Clodronate treatment had a minor effect on overall LAL activity in the liver tissue, despite the observed depletion of liver macrophages that contribute to hepatic LAL activity. The infiltration/proliferation of LAL-expressing CD68-positive macrophages increased due to HF/HCD feeding (Fig. 1C, D), and these cells are most likely responsible for the comparable overall activity of LAL in the livers of hepLal−/− mice (Fig. 4B, C). Co-immunofluorescence staining of LAL and CD68 revealed that LAL expression was uniformly distributed (most likely in hepatocytes) in WT liver sections due to the low abundance of macrophages, regardless of clodronate treatment (Fig. 4F). In the livers of hepLal−/− + PBS mice, the majority of LAL-positive cells were either CD68-positive or located near CD68-positive cells. Macrophage depletion markedly reduced the CD68-positive cells in hepLal−/− livers.

### 3.5 Hepatocyte LAL-D and KC depletion are associated with elevated circulating cholesterol levels

**Fig. 5:**
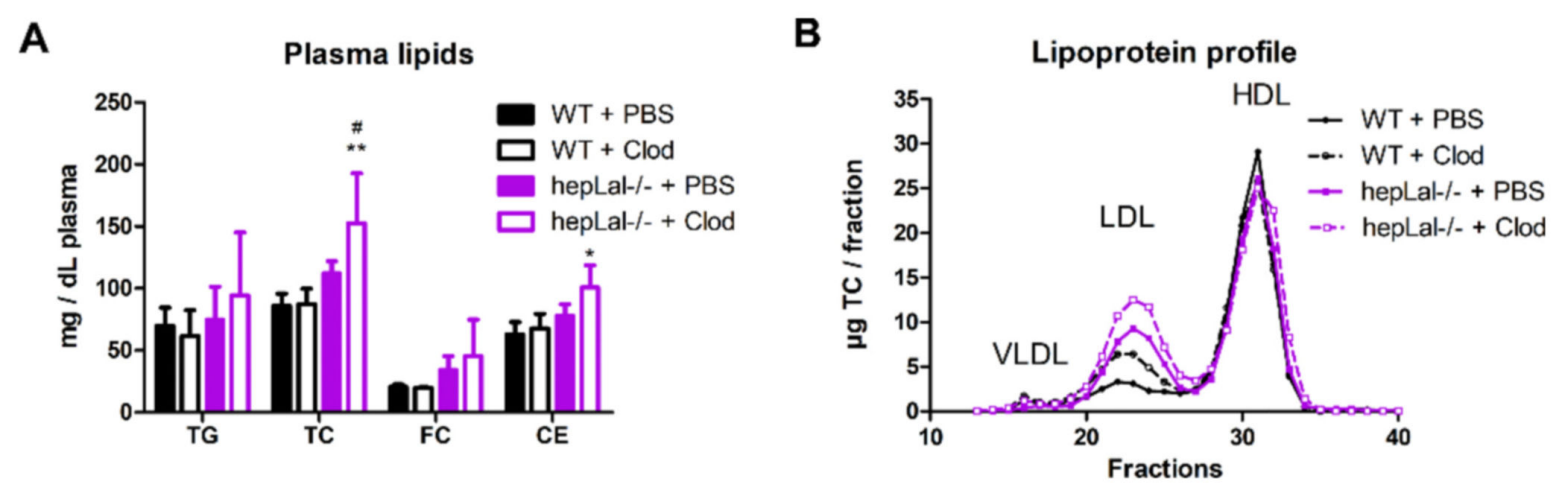
Hepatocyte-specific loss of LAL affects plasma lipid and lipoprotein profiles in HF/HCD-fed mice. Plasma and pWAT were isolated after 4 h of fasting from HF/HCD-fed WT and hepLal−/− mice injected i.p. with 3 doses of PBS- or clodronate (Clod)-liposomes. (A) Plasma TG, TC, FC, and CE concentrations (n = 3–6). (B) Lipoprotein profiles (very low-density lipoprotein (VLDL), low-density lipoprotein (LDL), high-density lipoprotein (HDL)) of TC after separation by fast performance liquid chromatography of pooled plasma (mean of two independent measurements with total pooled samples n = 4–6). Statistically significant differences were calculated by 2-way ANOVA followed by Tukey post-hoc test. *p < 0.05 and **p ≤ 0.01 for comparisons between different genotypes with the same treatment (WT + PBS vs hepLal−/− + PBS and WT + Clod vs hepLal−/− + Clod). p < 0.05 for comparisons between different treatments of the same genotypes (WT + PBS vs WT + Clod and hepLal−/− + PBS vs hepLal−/− + Clod).

Next, we examined the impact of hepatocyte LAL-D and KC depletion on plasma lipid parameters. The circulating TC concentrations tended to be elevated in hepLal−/− mice and reached significance following clodronate treatment. CE concentrations were increased in hepLal−/− compared to clodronate-treated WT mice. However, TG and FC levels remained unchanged (Fig. 5A). Low-density lipoprotein (LDL)-TC, which was already increased in the plasma of untreated hepLal−/− mice, was further aggravated by clodronate treatment. In contrast, very low-density lipoprotein (VLDL)-TC and high-density lipoprotein (HDL)-TC remained comparable (Fig. 5B). In contrast, untreated chow diet-fed WT and hepLal−/− mice had comparable lipoprotein profiles (Supplementary Fig. S2A), corroborating the fact that hepatocyte LAL plays a crucial role in lipoprotein metabolism only upon HF/HCD feeding. Overall, these data suggest that loss of LAL in hepatocytes of HF/HCD-fed mice, with or without KC depletion, affects plasma lipid and lipoprotein levels.

Neither hepatocyte-specific LAL-D nor clodronate treatment influenced the expression of lipid metabolism-related genes in pWAT of HF/HCD-fed mice. Despite repeated clodronate injections, the expression of macrophage markers remained unchanged in pWAT of both genotypes (Supplementary Fig. S2B). The mRNA expression of genes involved in lipid synthesis (*Acc, Fasn, Hmgcr, Agpat2, Gpat1, Dgat1, Dgat2*) (Supplementary Fig. S2C) and lipid degradation (*Pnpla2, Abhd5, Lipe, Mgll, Lipa*) (Supplementary Fig. S2D) were comparable between the genotypes, regardless of clodronate treatment. Consistent with these results, TG and CE hydrolase activities at pH 4.5 (Supplementary Fig. S2E, F) and pH 7 (Supplementary Fig. S2G, H) were unaltered. These data demonstrate that neither hepatocyte-specific LAL-D nor clodronate treatment affects lipid metabolism in pWAT. The changes observed in plasma parameters are most likely the consequence of macrophage depletion in the liver.

### 3.6 Liver proteomic analysis revealed significant proteome differences between genotypes, with minor changes observed between treatments

**Fig. 6:**
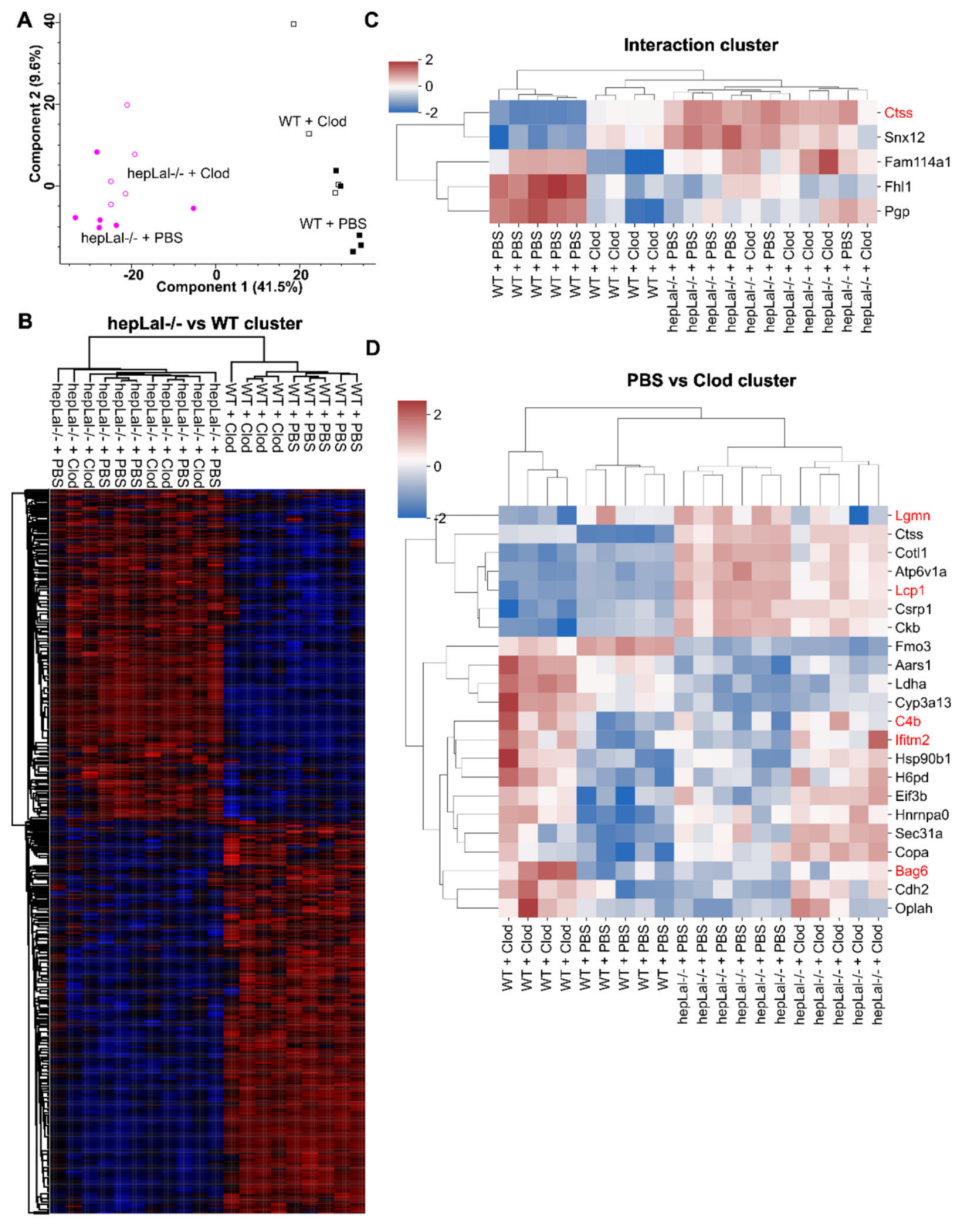
Hepatocyte-specific LAL-D triggers liver proteome changes in HF/HCD-fed mice. Livers were isolated from 4-h fasted WT and hepLal−/− mice injected i.p. with 3 doses of PBS- or clodronate (Clod)-liposomes. (A) Principal component analysis plot. Heatmap of z-scored significantly changed proteins between (B) hepLal−/− and WT, (C) interaction, and (D) PBS- vs Clod-treated mice. Proteins marked with red are associated with inflammation. Significantly changed proteins were determined with 2-way ANOVA and Benjamini-Hochberg FDR correction (FDR < 0.05). (For interpretation of the references to color in this figure legend, the reader is referred to the web version of this article.)

To investigate the effects of LAL-D and clodronate treatment on liver metabolism and inflammation in more detail, we performed a proteomic analysis. Principal component analysis revealed a clear separation between the liver proteomes of HF/HCD-fed WT and hepLal−/− mice (Fig. 6A). The less distinctive differences between PBS- and clodronate-treated livers supported the mRNA expression data, confirming the minor effect of KC depletion on the overall proteomic variation. We identified 1046 proteins that were significantly altered between the livers of HF/HCD-fed WT and hepLal−/− mice and showed similar expression patterns, irrespective of clodronate treatment (Fig. 6B). Conversely, only 5 proteins were significantly changed when the interaction between genotype and treatment was taken into account (Fig. 6C). Cathepsin S (CTSS), a lysosomal cysteine protease involved in inflammatory signaling [42], was the only significantly altered protein linked to the gene ontology biological process (GOBP) term containing “inflammatory”. Following clodronate treatment, CTSS expression decreased in WT livers but increased in hepLal−/− livers, whereas its overall expression remained higher in hepLal−/− livers compared to WT, regardless of treatment. Notably, CTSS was the only protein found to be significant across all three comparisons: genotype, treatment, and their interaction. Sorting nexin 12 (SNX12), a protein associated with endocytic degradative pathways [43], exhibited the same expression trend as CTSS. Glycerol-3-phosphate phosphatase (PGP) expression decreased in WT livers following clodronate treatment, while it remained comparable between PBS and clodronate-treated hepLal−/− livers. Significance testing identified 22 significantly altered proteins (Fig. 6D), with 5 of these proteins (legumain (LGMN), plastin-2 (LCP1), complement C4–B (C4B), interferon-induced transmembrane protein 2 (IFITM2), and large proline-rich protein BAG6 (BAG6)) associated with GOBP terms containing “leukocyte” or “cytokine.” LGMN and LCP1 exhibited lower expression in clodronate- compared to PBS-treated livers, whereas C4B, IFITM2, and BAG6 showed higher expression in clodronate-treated livers.

**Fig. 7:**
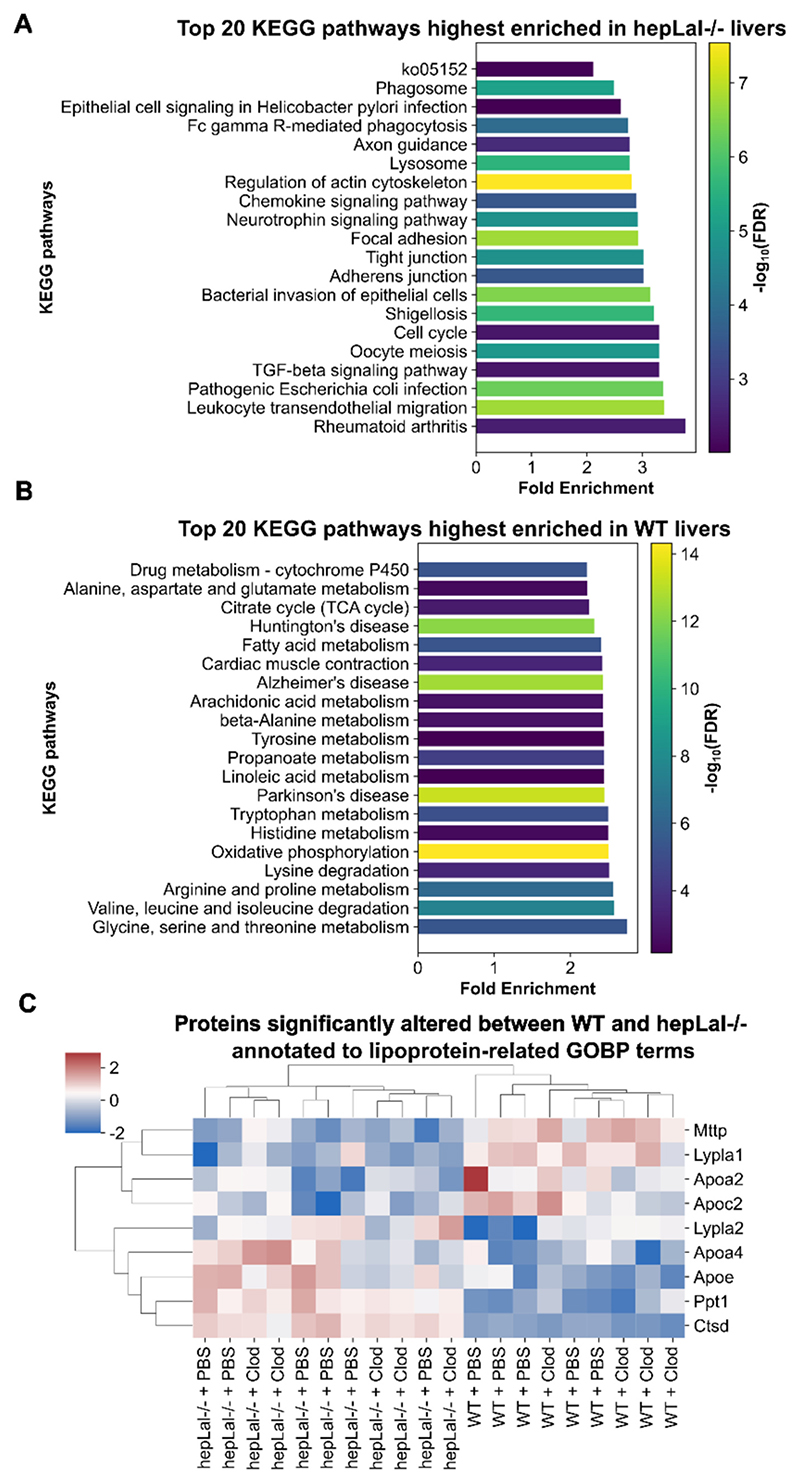
Hepatocyte-specific LAL-D dysregulates multiple lipid metabolism-related KEGG terms and impairs expression of proteins involved in lipoprotein metabolism in HF/HCD-fed mice. Top 20 KEGG pathways highest enriched in livers of (A) hepLal−/− and (B) WT mice. (C) Heatmap showing z-scored intensities of significantly changed proteins between hepLal−/− and WT livers annotated to lipoprotein-related gene ontology biological process (GOBP) terms. Significance was determined with (A, B) Fisher exact test and Benjamini-Hochberg FDR correction set at under 0.02 or with (C) 2-way ANOVA and Benjamini-Hochberg FDR correction (FDR < 0.05).

To explore the dysregulated processes driven by the major proteome changes observed in WT vs hepLal−/− livers, we performed KEGG pathway analysis. In hepLal−/− livers, the highest enriched KEGG pathways of significantly altered proteins indicated upregulation of lysosomal proteins and pathways related to inflammation, such as leukocyte transendothelial migration, chemokine signaling, and Fc gamma R-mediated phagocytosis, as well as fibrosis-related pathways, including TGF-beta signaling (Fig. 7A). In contrast, the top 20 KEGG pathways downregulated in hepLal−/− livers included those related to lipid metabolism (linoleic, arachidonic, fatty acid metabolism) and energy metabolism (oxidative phosphorylation, citrate cycle, and metabolism of essentially all amino acids) (Fig. 7B). Since lipoprotein profiles are altered in hepLal−/− mice (Fig. 5B), we explored which lipoprotein metabolism-related proteins were significantly changed between WT and hepLal−/− livers. Microsomal triglyceride transfer protein (MTTP), apolipoprotein A-II (APOA2), and APOC2 were reduced in hepLal−/− livers compared to WT regardless of treatment, indicating diminished VLDL and HDL synthesis and LPL activation (Fig. 7C). In contrast, the expression of APOA4 and APOE was higher in hepLal−/− compared to WT livers, suggesting a complex dysregulation of lipoprotein metabolism. Consistent with the LAL protein expression (Fig. 4A) and activity (Fig. 4B and C), proteomic analysis also revealed no change in LAL abundance (Supplementary Table S2), most likely due to the prevalence of infiltrating macrophages in the livers of HF/HCD-fed hepLal−/− mice.

### 3.7 Hepatocytes isolated from HF/HCD-fed hepLal−/− mice exhibited impaired lipid metabolism

**Fig. 8:**
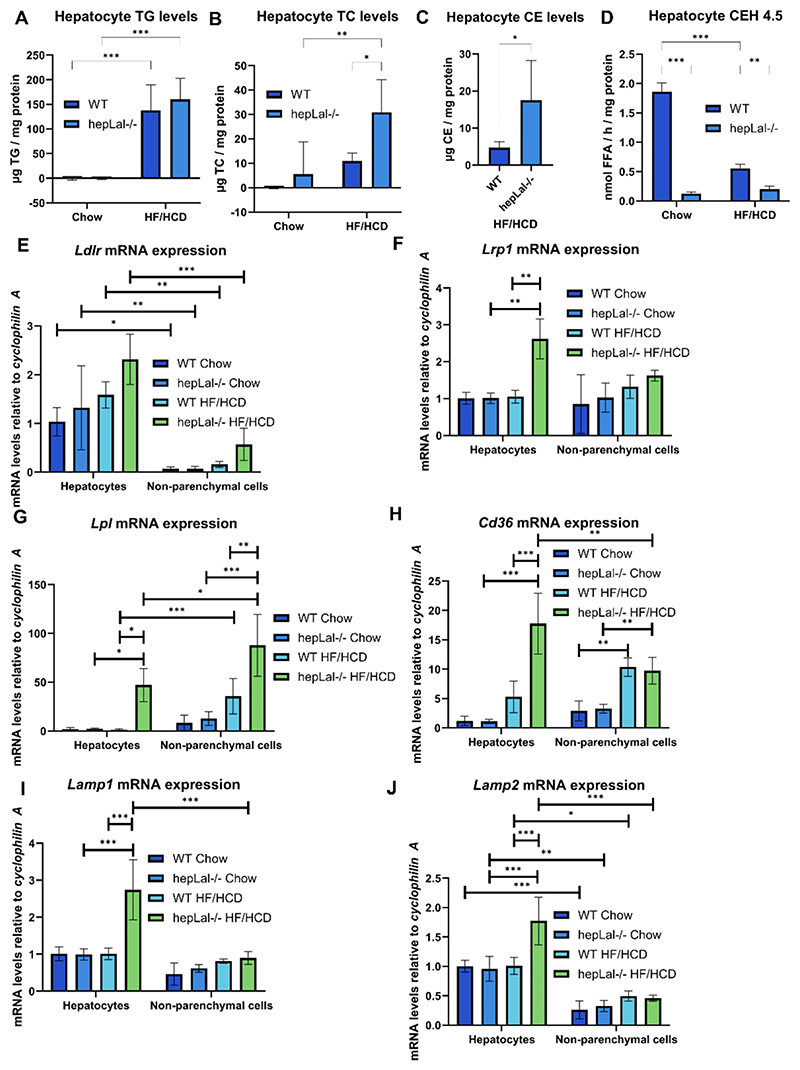
Hepatocytes from HF/HCD-fed hepLal−/− mice accumulate cholesterol and have impaired expression of genes involved in lipid transport and degradation. Hepatocyte- and non-parenchymal cell-enriched fractions were isolated from WT and hepLal−/− mice fed either chow or HF/HCD. Quantification of (A) TG, (B) TC, and (C) CE concentrations (n = 5). (D) Hepatocyte CE hydrolase (CEH) activity at pH 4.5 (n = 3–5). (E–J) mRNA expression of lipid metabolism-related genes in hepatocyte- and non-parenchymal cell-enriched fractions (n = 3–5). Statistically significant differences were calculated by (A, B, D) 2-way ANOVA followed by Tukey post-hoc test, (C) 2-tailed Student’s *t*-tests, or (E–J) 3-way ANOVA followed by Tukey post-hoc test. *p < 0.05, **p ≤ 0.01, and ***p ≤ 0.001.

To further scrutinize lipid metabolism specifically in hepatocytes, we isolated hepatocytes and NPC from chow- and HF/HCD-fed WT and hepLal−/− mice. mRNA expression of the hepatocyte marker *G6pase* confirmed the successful hepatocyte isolation (Supplementary Fig. S3A). In contrast, the NPC markers *Emr1, Cd31, Clec4f*, and *Cd68* were almost exclusively expressed in the NPC fraction (Fig. S3B-E). Following HF/HCD feeding, NPCs from WT and even more from hepLal−/− mice exhibited a markedly increased expression of *Cd68* (Fig. S3E), consistent with the hypothesis that CD68-positive macrophages increase in the liver following a HF/HCD challenge. Hepatocyte TG concentrations were significantly elevated in both HF/HCD-fed WT and hepLal−/− mice compared to chow-fed mice (Fig. 8A). In addition, hepatocyte TC content was higher in HF/HCD-fed hepLal−/− than in chow-fed hepLal−/− mice and HF/HCD-fed WT mice (Fig. 8B), and CE levels were increased in HF/HCD-fed hepLal−/− mice compared to WT mice (Fig. 8C). CEH activity in the hepatocytes at pH 4.5, resembling LAL activity, was significantly reduced in hepLal / mice in both feeding conditions (Fig. 8D). Moreover, HF/HCD decreased LAL activity in WT hepatocytes (Fig. 8D). *Ldlr* expression was comparable between WT and hepLal−/− mice in both hepatocytes and NPCs (Fig. 8E), suggesting that no major transcriptional regulation of lipoprotein uptake occurs in hepLal−/− mice. Elevated hepatic *Lrp1* mRNA expression in HF/HCD-fed hepLal−/− compared to both HF/HCD-fed WT and chow-fed hepLal−/− mice (Fig. 8F) may suggest increased uptake of APOE-containing lipoproteins. Increased *Lpl* and *Cd36* levels in HF/HCD-fed hepLal−/− hepatocytes compared to both HF/HCD-fed WT and chowfed hepLal−/− mice (Fig. 8G, H) suggested increased TG hydrolysis from lipoproteins and enhanced uptake of fatty acids. Increased TG hydrolysis from VLDL may explain the elevated LDL levels observed in HF/HCD-fed hepLal−/− mice (Fig. 5B). Increased mRNA expression levels of the lysosomal markers *Lamp1* and *Lamp2* in hepatocytes of HF/HCD-fed hepLal−/− mice (Fig. 8I, J) suggest lysosomal accumulation to compensate for an impaired lysosomal function.

## 4 Discussion

The growing evidence points to an important role of LAL and macrophages [40] in the pathogenesis of MASLD. Recent findings suggested that circulating LAL activity inversely correlates with MASLD severity [44]. In light of these findings, we analyzed hepatic lipid metabolism in HF/HCD-fed hepLal−/− mice with or without KC depletion. Fed a chow diet, hepLal−/− mice accumulate CE in the liver but lack significant alterations in genes related to hepatic lipid homeostasis [26,45]. When challenged with a HF/HCD, hepLal−/− mice exhibit impaired glucose metabolism, dyslipidemia, and profound liver injury and inflammation [26]. In addition to hepatomegaly, we observed that these mice develop splenomegaly, which was also demonstrated in MASLD patients with reduced circulating LAL activity in the absence of advanced chronic liver disease and portal hypertension [46]. We attempted to measure LAL activity in mouse blood following the dried-blood spot method developed for human samples [47]. However, pronounced LAL activity in the blood of Lal−/−mice prevented a reliable estimation of circulating LAL levels, hindering a direct correlation of hepatic LAL deficiency with systemic enzyme activity. Clodronate-mediated depletion of KC neither affected body nor tissue weights of either genotype. The livers of hepLal−/− mice showed decreased expression of genes involved in lipid synthesis, transport, and degradation as well as fatty acid oxidation, which were all largely unaltered by clodronate treatment. Proteome changes were mostly in line with the observed mRNA changes. Since we determined mRNA and protein expression in bulk liver tissue, we cannot exclude the possibility that the observed expression changes may result from alterations in the distribution of the subpopulations.

Depletion of LAL-expressing macrophages in hepLal−/− mice increased TC and CE levels in the liver. Interestingly, lower PGP levels observed in hepLal−/− livers regardless of the treatment are linked to enhanced TG synthesis and reduced fatty acid oxidation, whereas higher PGP levels are known to decrease CE concentrations in primary hepatocytes [42]. As both LAL [15] and KC [48] are critical for LDL degradation, the increase in circulating LDL-TC concentrations and an additional increase upon KC depletion were expected. Despite unchanged *Ldlr* mRNA expression in isolated hepatocytes and NPCs, several proteins involved in lipoprotein metabolism were dysregulated in hepLal−/− livers (MTTP, APOA2, APOC2, APOA4, APOE) and *Lpl* and *Cd36* mRNA expression were increased in hepatocytes, suggesting that VLDL synthesis and VLDL-TG hydrolysis might be affected in hepatic LAL–D. This finding could contribute to increased LDL levels, especially when endolysosomal function is compromised, as in LAL–D. In general, *Lpl* is expressed at very low levels in the livers of adult mice; however, hepatic deletion of *Lpl* results in decreased plasma LPL concentrations and impaired plasma TG clearance [49]. Our results suggest that i.p. injected clodronate primarily targets liver resident macrophages, as there were no effects of hepatocyte LAL-D and clodronate treatment on macrophage- and inflammation-related genes in pWAT. The unaltered mRNA expression of genes involved in lipid synthesis and hydrolysis, as well as unchanged lipase activities in pWAT, exclude the possibility that the observed changes in liver and plasma lipid metabolism are a consequence of impaired lipid metabolism in WAT.

Despite decreased *Lipa* mRNA expression, acidic CE and TG hydrolase activities and LAL protein levels were comparable in the liver of both genotypes when fed a HF/HCD. These observations contradict the previous findings of decreased acid CE and TG hydrolase activities in chow diet-fed hepLal−/− mice [41]. However, the unchanged acidic CE and TG hydrolase activities in HF/HCD-fed hepLal−/− mice might occur due to the massive infiltration of LAL-expressing macrophages. This hypothesis is supported by the fact that (i) macrophage-specific LAL expression in mice with global LAL-D significantly reduces CE and TG levels in the liver [50], (ii) LAL activity was decreased in hepatocytes isolated from HF/HCD-fed hepLal−/− mice, and (iii) LAL was co-expressed with CD68 in the liver of these mice. Reduced LAL activity correlates with MASLD [11,51,52], so decreased LAL activity in HF/HCD-fed WT mice might hint at MASLD development. Clodronate treatment depleted *Clec4f*-expressing KC but failed to deplete all macrophages in hepLal−/− livers. This may be caused by the massive infiltration/proliferation of CD68-positive cells or the impairment of the phagocytic function of macrophages, as demonstrated in MASH mouse [53] and rat models and in macrophages from MASLD patients [54].

Elevated WBC counts suggest that hepatocyte LAL-D is associated with systemic inflammation. KC and infiltrating monocytes/macrophages promote the development of MASLD and the progression to MASH and fibrosis, particularly in the context of HF/HCD feeding [40]. Conversely, infiltrating macrophages may also exhibit tissue remodeling and/or antifibrotic properties [55]. The mechanism by which KC may trigger the development of MASH includes the following: (i) the accumulation of cholesterol in hepatocytes and the formation of cholesterol (ester) crystals, which eventually lead to hepatocyte death; (ii) KC subsequently may surround the dead hepatocytes and hydrolyze CE remnants, leading to (iii) increased inflammatory signaling and MASH [56]. The absence of liver fibrosis in control mice even after 14 weeks of HF/HCD was consistent with previous data showing that only feeding a HF/HCD for 30 weeks, but not for 12 weeks, caused liver fibrosis in WT mice [28]. In contrast, despite remaining lean, HF/HCD-fed hepLal−/− mice were prone to developing liver fibrosis, as evidenced by increased expression of multiple markers of liver fibrosis, including *Mmps* and *Timps* [57], collagen content, and Sirius red staining. Although MASLD is often associated with obesity, up to 20 % of patients are lean [58]. It is elusive whether LAL activity might be a contributing factor to the development of MASLD in individuals with a lean phenotype. At least in mice challenged with HF/HCD, decreased LAL activity in hepatocytes is sufficient to trigger liver inflammation and macrophage infiltration.

The depletion of KC by clodronate resulted in a reduction in the mRNA expression of *Mmps, Timps*, fibrosis-related markers, and macrophage-related genes in the livers of hepLal−/− mice. An improvement of hepatic steatosis and inflammation by KC depletion was also observed in other mouse [31,32,59] and rat models [60] of MASLD. However, two studies indicated no significant [61] or even negative effects [33] of KC depletion on liver steatosis and inflammation. We observed that short-term depletion of liver macrophages through clodronate treatment failed to alter collagen levels in hepLal−/− mice, indicating that acute targeted removal of resident macrophages may not be sufficient to improve an already injured liver. In addition, lysosomal lipid hydrolysis in macrophages provides precursor molecules for critical lipid mediators [62], suggesting that removing macrophages (infiltrating and/or resident ones) may have adverse effects on MASLD. Despite a reduction in the mRNA expression of fibrosis-related genes, ALT and AST levels were unaffected by clodronate treatment. It is expected that a pronounced remodeling of the liver with less damage would take much longer than one week of clodronate treatment.

KEGG pathway analysis of proteins upregulated in hepLal−/− livers revealed increased leukocyte migration, phagocytosis, and chemokine signaling, which demonstrated pronounced inflammation. Clodronate treatment reduced the expression of LGMN, a protein involved in the degradation of fibronectin, which typically increases during the differentiation of monocytes into macrophages [63,64]. These results imply that clodronate may promote an environment favorable for fibrosis development, potentially explaining why collagen staining remained unchanged despite the downregulation of mRNA expression of fibrosis-related genes. LCP1, which plays a crucial role in the regulation of inflammatory cell migration [65], was similarly downregulated by clodronate. However, given that LCP1 activity is mainly controlled through phosphorylation [65], alterations in expression levels may not reflect its functional implications. C4B, IFITM2, and BAG6 were among the inflammation-related proteins whose expression increased following clodronate treatment. Upregulated expression of C4B indicated elevated phagocytosis of pathogens and dead or dying cells [66], suggesting the removal of dead macrophages by other cells after clodronate treatment. Since hepatocytes are also competent efferocytes [67], their ability to capture dying macrophages might also explain restored LAL activity in hepLal−/− livers. Clodronate injection led to elevated IFITM2 levels in both genotypes. Since IFITM2 is expressed by T cells and plays a role in their differentiation into T helper 2 cells [68], T cells may be mobilized to support tissue repair after macrophage depletion [69]. Although we did not detect specific markers of other immune cells in the proteomic analysis, increased expression of BAG6 after clodronate treatment indicated changes in natural killer (NK) cell activation [70]. Inhibition of CTSS also reduces NK cell expansion [42] and decreases NF-κB-dependent hepatic inflammation [71]. Thus, increased CTSS levels in hepLal−/− livers may implicate enhanced NK cell expansion and hepatic inflammation. Clodronate treatment slightly decreased CTSS levels in hepLal−/− livers, consistent with the fact that macrophages are a major hepatic source of CTSS [72]; nevertheless, other liver cells also express significant amounts of CTSS [71]. Cathepsins, usually found in endolysosomal compartments, are also involved in the development of lysosomal storage disorders [73]. The deficiency of LAL in hepatocytes results in massive infiltration of macrophages and lipid buildup in lysosomes, impairing their function [26]. As a result, macrophages may account for most of the hepatic CTSS expression in hepLal−/− mice. In contrast, clodronate treatment of WT mice resulted in higher CTSS abundance, and hepatocytes are likely the primary source of CTSS due to less infiltrating macrophages. Based on proteomic analysis, we conclude that LAL-D in hepatocytes triggered significant proteome changes, leading to the enrichment of multiple inflammation-related KEGG terms. In contrast, clodronate treatment had a milder and more specific effect, affecting only a limited number of proteins, some of which play a key role in regulating the inflammatory response. Of note, the hepatic immune landscape was not characterized directly, e.g. by flow cytometry, but rather assessed indirectly based on proteomic data. Several compounds that act on macrophages, primarily by inhibiting inflammatory processes, are currently being tested as attractive therapeutic targets for treating MASLD, however, with varying degrees of success [40]. Therefore, it is recommended to explore the potential treatment option of modulating inflammation instead of depleting macrophages in liver pathologies with reduced LAL activity.

We conducted the experiments exclusively in female mice. Whether male mice may have responded differently to the clodronate treatment remains to be investigated. Nevertheless, our data demonstrate that the loss of LAL in hepatocytes leads to the accumulation of CE, macrophage infiltration, inflammation, and fibrosis in the livers of female mice fed a HF/HCD. The depletion of KC causes an aggravation of liver and plasma cholesterol levels with minor effects on inflammation and no significant impact on fibrosis in hepLal−/− mice. Decreasing the inflammatory phenotype of macrophages rather than depletion of these cells may therefore be a more viable treatment to reduce liver fibrosis without compromising lipoprotein metabolism.

## Supplementary Material

Supplementary data to this article can be found online at https://doi.org/10.1016/j.bbalip.2024.159575.

suppl figures and Table S1

Suppl Table S2

## Data Availability

Data will be made available on request.

## Abbreviations

ALT: alanine aminotransferase
Apo: apolipoprotein
AST: aspartate aminotransferase
BAT: brown adipose tissue
BAG6: large proline-rich protein
C4B: complement C4-B
CE: cholesteryl ester
CEH: cholesteryl ester hydrolase
CTSS: cathepsin S
FC: free cholesterol
GOBP: gene ontology biological process
HDL: high-density lipoprotein
HF/HCD: high fat/high cholesterol diet
IFITM2: interferon-induced transmembrane protein 2
KC: Kupffer cells
LAL: lysosomal acid lipase
LAL-D: lysosomal acid lipase deficiency
LCP1: platin-2
LDL: low-density lipoprotein
LGMN: legumain
MASLD: metabolic dysfunction-associated steatotic liver disease
MASH: metabolic dysfunction-associated steatohepatitis
MTTP: microsomal triglyceride transfer protein
NPC: non-parenchymal cells
PGP: glycerol-3-phosphate phosphatase
SNX12: sorting nexin 12
sWAT: subcutaneous white adipose tissue
TC: total cholesterol
TG: triacylglycerol
TGH: triacylglycerol hydrolase
pWAT: perigonadal white adipose tissue
VLDL: very low-density lipoprotein
WBC: white blood cells
WT: wild-type
