# Supplementary material for: From LAL-D to MASLD: Insights into the role of LAL and Kupffer cells in liver inflammation and lipid metabolism
Source: Biochim Biophys Acta Mol Cell Biol Lipids. Author manuscript; Available in PMC 2026 Jan 16. (PMC7618626; doi:10.1016/j.bbalip.2024.159575)
Supplement: suppl figures and Table S1 [file EMS212066-supplement-suppl_figures_and_Table_S1.pdf]

Supplementary Figures and Table S1

From LAL-D to MASLD: Insights into the role of LAL and Kupffer cells in liver inflammation and lipid metabolism

Ivan Bradić, Katharina B. Kuentzel, Anita Pirchheim, Silvia Rainer, Birgit Schwarz, Michael Trauner, Martin R. Larsen, Nemanja Vujić, and Dagmar Kratky

Figure S1

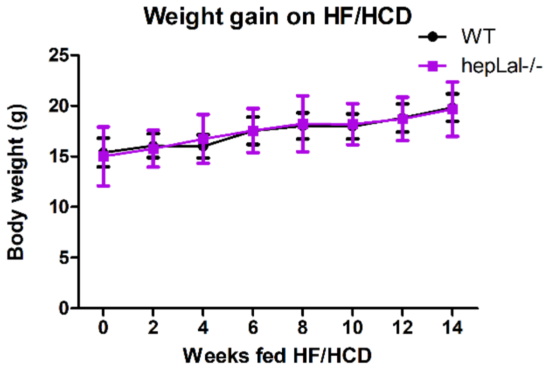

Figure S1. HF/HCD-fed hepLal-/- mice show comparable weight gain as WT mice. Body weight of WT and hepLal-/- mice fed HF/HCD for 14 weeks (n = 7).

Figure S2

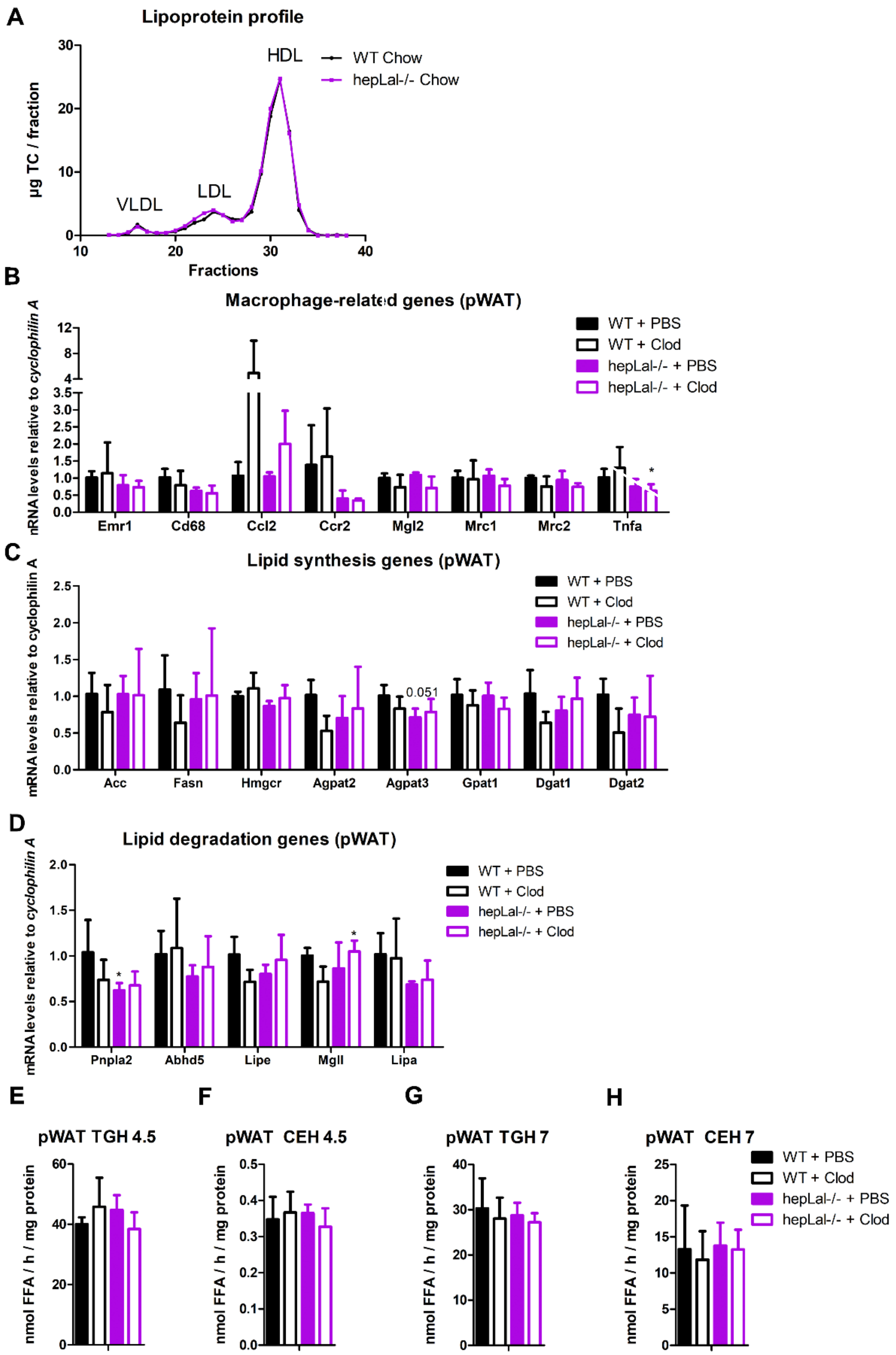

A
Lipoprotein profile
µg TC / fraction
Fractions
VLDL
LDL
HDL
WT Chow
hepLal-/- Chow
B
Macrophage-related genes (pWAT)
mRNA levels relative to cyclophilin A
Emr1
Cd68
Ccl2
Ccr2
Mgl2
Mrc1
Mrc2
Tnfa
WT + PBS
WT + Clod
hepLal-/- + PBS
hepLal-/- + Clod
C
Lipid synthesis genes (pWAT)
mRNA levels relative to cyclophilin A
Acc
Fasn
Hmgcr
Agpat2
Agpat3
Gpat1
Dgat1
Dgat2
0.051
D
Lipid degradation genes (pWAT)
mRNA levels relative to cyclophilin A
Pnpla2
Abhd5
Lipe
Mgll
Lipa
E
pWAT TGH 4.5
F
pWAT CEH 4.5
G
pWAT TGH 7
H
pWAT CEH 7
nmol FFA / h / mg protein

Figure S2: Lipid-associated mRNA expression and LAL activity are unaffected in pWAT of hepLal-/- mice. (A) Lipoprotein profiles of chow diet-fed WT and hepLal-/- mice. TC concentrations were determined in very low-density lipoprotein (VLDL), low-density lipoprotein (LDL), and high-density lipoprotein (HDL) fractions after separation by fast performance liquid chromatography of pooled plasma (n = 4). pWAT was isolated after 4 h of fasting from HF/HCD-fed WT and hepLal-/- mice injected i.p. with 3 doses of PBS- or clodronate (Clod)-liposomes. mRNA expression of genes related to (B) macrophages, (C) lipid synthesis, and (D) lipid degradation (n = 4 – 6). (E, G) TG hydrolase (TGH) and (F, H) CE hydrolase (CEH) activities at (E, F) pH 4.5 and (G, H) pH 7 (n = 4). Statistically significant differences were calculated by 2-way ANOVA followed by Tukey post-hoc test. *$p < 0.05$ for comparisons between different genotypes with the same treatment (WT + PBS vs hepLal-/- + PBS and WT + Clod vs hepLal-/- + Clod).

Figure S3

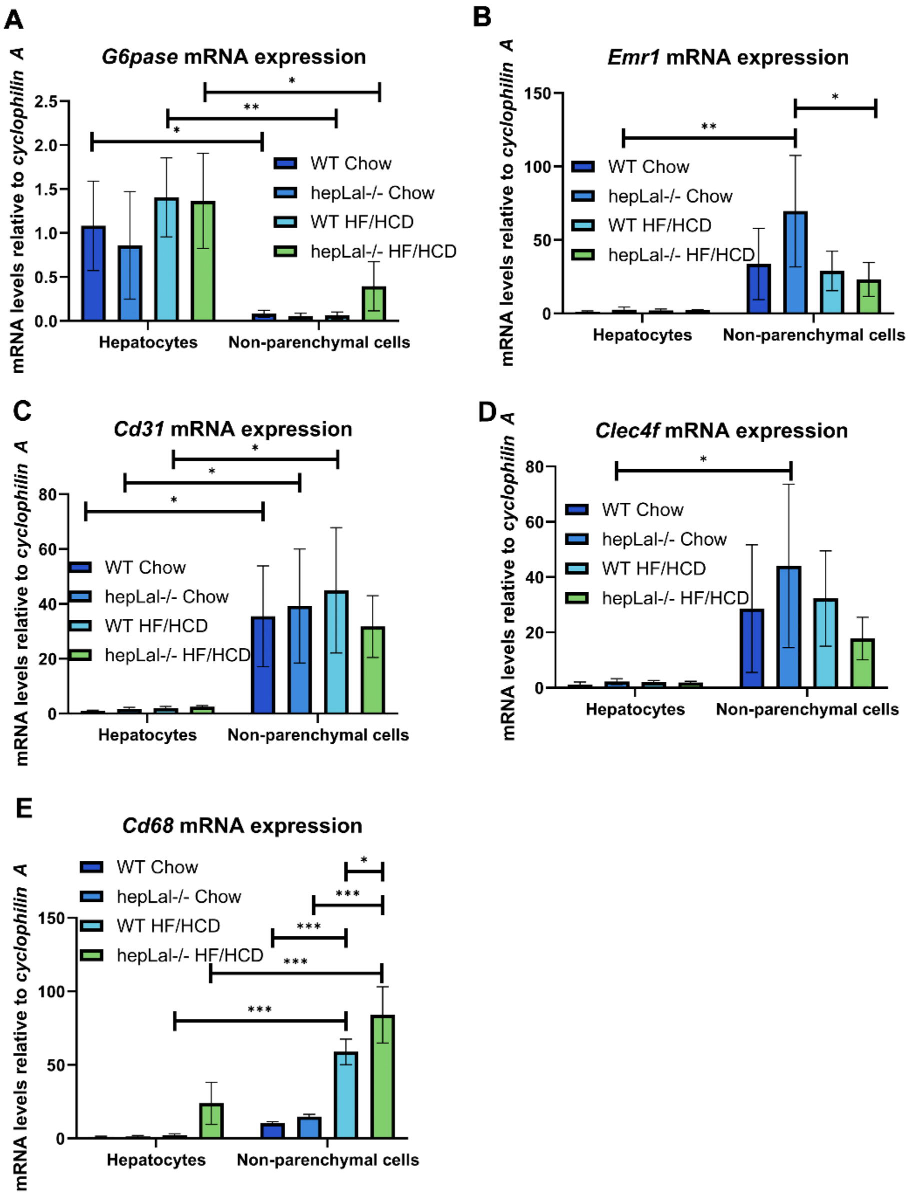

Figure S3: qPCR results as confirmation of successful hepatocyte- and non-parenchymal cell-enriched fractions. (A-E) mRNA expression of hepatocyte and non-parenchymal cell markers in hepatocyte- and non-parenchymal cell-enriched fraction (n = 3 – 5). Statistically significant differences were calculated by 3-way ANOVA followed by Tukey post-hoc test. *$p < 0.05$, **$p \leq 0.01$, and ***$p \leq 0.001$.

Table S1. Primer sequences

Gene	Forward Sequence 5' – 3'	Reverse Sequence 5' – 3'
Abhd5	GGTTAAGTCTAGTGCAGC	AAGCTGTCTCACCACTTG
Acadm	AGGGTTTAGTTTTGAGTTGACGG	CCCCGCTTTTGTCATATTCCG
Acc	GGACTTGGAGCAGAGAACCTTCG	CAAGCTGGTTGTTGGAGGTGTA
Acox1	TCCAGACTTCCAACATGAGGA	CTGGGCGTAGGTGCCAATTA
Acsl1	ACCAGCCCTATGAGTGGATTT	CAAGGCTTGAACCCCTTCTG
Acta2	GGACGTACAACTGGTATTGTGC	TCGGCAGTAGTCACGAAGGA
Agpat2	CAGCCAGGTTCTACGCCAAG	TGATGCTCATGTTATCCACGGT
Agpat3	CTGCTTGCCTACCTGAAGACC	GATACGGCGGTATAGGTGCTT
Ccl2	TTAAAAACCTGGATCGGAACCAA	GCATTAGCTTCAGATTTACGGGT
Ccr2	ATCCACGGCATACTATCAACATC	CAAGGCTCACCATCATCGTAG
Cd36	GCAGGTCTATCTACGCTGTG	GGTTGTCTGGATTCTGGAGG
Cd68	AACAGGACCTACATCAGAGC	TCAAGGTGAACAGCTGGAGA
Clec4f	CCCAAGGACTGGGGCCTAA	AGGGAGGCTCTTTATTCCACT
Col1a1	TAAGGGTCCCCAATGGTGAGA	GGGTCCCTCGACTCCTACAT
Col1a2	TCGTGCCTAGCAACATGCC	TTTGTCAGAATACTGAGCAGCAA
Cpt1a	CTCCGCCTGAGCCATGAAG	CACCAGTGATGATGCCATTCT
Dgat1	TCCGCCTCTGGGCATTC	GAATCGGCCCACAATCCA
Dgat2	AGTGGCAATGCTATCATCATCGT	TCTTCTGGACCCATCGGCCCCAGGA
Emr1	CTTTGGCTATGGGCTTCCAGTC	GCAAGGAGGACAGAGTTTATCGTG
Fasn	GAAGCCGAACACCTCTGTGCAGT	GCTCCTTGCTGCCATCTGTATTG
Gpat1	ACAGTTGGCACAATAGAGGTTT	CCTTCCATTTCAGTGTTGCAGA
Hmgcr	TGTTCACCGGCAACAACAAGA	CCGCGTTATCGTCAGGATGA
Ldlr	CTCCTGCATTCACGGTAGCC	CCCACTGTGACACTTGAACTTG
Lipa	GGATGAGTTCTGGGCCTTCAG	AAACCTATGGTGCAGCCTTGAG
Lipe	GATTTACGCACGATGACACAGT	ACCTGCAAAGACATTAGACAGC
Lrp1	ACTATGGATGCCCCTAAAACTTG	GCAATCTCTTTCACCGTCACA
Mgl2	CAATGTGCTTAGCTGGATGGG	CCATGCCAGTTATCCGGCTG
Mgll	CGGACTTCCAAGTTTTTGTCAGA	GCAGCCACTAGGATGGAGATG
Mmp12	CTGCTCCCATGAATGACAGTG	AGTTGCTTCTAGCCCAAAGAAC
Mmp13	CTTCTTCTTGTTGAGCTGGACTC	CTGTGGAGGTCACTGTAGACT
Mmp14	CAGTATGGCTACCTACCTCCAG	GCCTTGCCTGTCACTTGTAAA
Mmp19	CCTGGTCCCATGCCAAACC	CCCTTGAAAGCATAAGTCTTCCC
Mmp1a	CCTTGATGAGACGTGGACCAA	ATGTGGTGTTGTTGCACCTGT
Mmp2	ACCTGAACACTTTCTATGGCTG	CTTCCGCATGGTCTCGATG
Mrc1	GCTGAATCCCAGAAATTCCGC	ATCACAGGCATACAGGGTGAC
Mrc2	TCTCCCGGAACCGACTCTTC	AACTGGTCCCCTAGTGTACGA
Pnpla2	GCCACTCACATCTACGGAGC	GACAGCCACGGATGGTGTTC
Ppara	TTCACAAGTGCCTGTCTGTC	GGCCTTGACCTTGTTCATGT
Ppargc1a	TTCATCTGAGTATGGAGTCGCT	GGGGGTGAAACCACTTTTGTAA

Timp1	GCAACTCGGACCTGGTCATAA	CGGCCCGTGATGAGAAACT
Timp2	CTCGCTGTCCCATGATCCC	GCCCATTGATGCTCTTCTCTGT
Timp3	CTTCTGCAACTCCGACATCGT	GGGGCATCTTACTGAAGCCTC
Tnfa	ATTCGAGTGACAAGCCTGTAGC	GGTTGTCTTTGAGATCCATGCC
Lpl	ACATTCCCGTTACCGTCCATC	AGGACCCCTGAAGACACAG
Lamp1	CAGCACTCTTTGAGGTGAAAAAC	CCATTCGCAGTCTCGTAGGTG
Lamp2	ATGTGCCTCTCTCCGGTTAAA	GCAAGTACCCTTTGAATCTGTCA
